# Supplementary material for: Advancing lignin analytics via elucidation of linkage progressions in lignin populations
Source: Commun Chem. 2025 Dec 11;9:31. doi: 10.1038/s42004-025-01841-3 (PMC12820315; doi:10.1038/s42004-025-01841-3)
Supplement: Supplementary file 2 — Supplementary Information [file 42004_2025_1841_MOESM2_ESM.pdf]

Supporting information

Advancing Lignin Analytics via Elucidation of Linkage Progressions in Lignin Populations

Filippa Ludvig[1,2], Åsa Emmer[3], Martin Lawoko[1,2*]

[1] WWSC Wallenberg Wood Science Center, School of Engineering Sciences in Chemistry, Biotechnology and Health, KTH Royal Institute of Technology, 100 44 Stockholm, Sweden
[2] Division of Wood Chemistry and Pulp Technology, Department of Fibre and Polymer Technology, School of Engineering Sciences in Chemistry, Biotechnology and Health, KTH Royal Institute of Technology, 100 44 Stockholm, Sweden
[3] Applied Physical Chemistry, Department of Chemistry, School of Engineering Sciences in Chemistry, Biotechnology and Health, KTH Royal Institute of Technology, 100 44 Stockholm, Sweden

* Corresponding author email address: lawoko@kth.se

Table of figures

Supplementary Figure 1. MALDI-TOF spectra of pure 2,5-DHB matrix. 20 mg ml^{-1} in TA30+ 0.1% TFA. 0-5000 m/z was investigated. The peaks shown are those stemming from the pure matrix. Y-axis display signal intensity, and x-axis display mass-over-charge (m/z) value. Above approximately 831 m/z, no signal from the matrix is recorded. 5

Supplementary Figure 2. MALDI-TOF MS spectra of ENZDHP. 0-5000 m/z was investigated. Y-axis display signal intensity, and x-axis display mass-over-charge (m/z) value. Clusters are representative of one monomer adding into the chain, which gives a spread in m/z given that the addition of a monomer with or without water and different connectivity yields different m/z increments. Weak clusters can be seen up to around 2500 m/z, but more intense clusters are seen until around 2000 m/z. 6

Supplementary Figure 3. MALDI-TOF MS spectra of FEDHP. 0-5000 m/z was investigated. Y-axis display signal intensity, and x-axis display mass-over-charge (m/z) value. Clusters are representative of one monomer adding into the chain, which gives a spread in m/z given that the addition of a monomer with or without water and different connectivity yields different m/z increments. Weak clusters are seen until approximately 2000 m/z, and stronger clusters are recorded up until around 1500 m/z. 7

Supplementary Figure 4. MALDI-TOF MS spectra of MWLS. 0-5000 m/z was investigated. Y-axis display signal intensity, and x-axis display mass-over-charge (m/z) value. Clusters are representative of one monomer adding into the chain, which gives a spread in m/z given that the addition of a monomer with or without water and different connectivity yields different m/z increments. Clusters are seen up until 1700 m/z, with a broad slope tailing out around 2000 m/z. 8

Supplementary Figure 5. MALDI-TOF MS spectra of acetylated FEDHP (ACFEDHP). Spectra were acquired for 0-5000 m/z but to give a better overview of regions were acetylated m/z adducts are actually present, the spectra are zoomed in to 400-2200 m/z. Notice the m/z for acetylated β-O-4'-dimer (567 m/z) being among the most intense peaks in all spectra. Y-axis display signal intensity, and x-axis display mass-over-charge (m/z) value. Clusters are representative of one monomer adding into the chain, which gives a spread in m/z given that the addition of a monomer with or without water and different connectivity yields different m/z increments. Marked distances for the respective acetylated increments discussed in the article are marked, and 442 m/z is also found in the sample. 9

Supplementary Figure 6. MALDI-TOF MS spectra of acetylated MWLS (MWLSA) obtained from inner crystallization area on MALDI-TOF MS sample spots. 0-5000 m/z was investigated. Y-axis display signal intensity, and x-axis display mass-over-charge (m/z) value. Clusters are representative of one monomer adding into the chain, which gives a spread in m/z given that the addition of a monomer with or without water and different connectivity yields different m/z increments.. 10

Supplementary Figure 7. MALDI-TOF MS spectra of acetylated MWLS (MWLSA) obtained from outer crystallization area on MALDI-TOF MS sample spots. 0-5000 m/z was investigated. Descriptive picture of crystallization regions is inserted underneath MALDI-TOF spectra. In spectra, Y-axis display signal intensity, and x-axis display mass-over-charge (m/z) value. Clusters are representative of one monomer adding into the chain, which gives a spread in m/z given that the addition of a monomer with or without water and different connectivity yields different m/z increments.. 11

Supplementary Figure 8. MALDI-LIFT-TOF/TOF MS^2 of MWLSA with HCCA matrix (bottom) and pure HCCA matrix (top) when collecting 1080.9070 m/z. Y-axis display signal intensity, and x-axis display mass-over-charge (m/z) value. Counting from top, 2nd and 4th spectra are parent spectra, while 1st and 3rd are fragment spectra. Most important note

here is the fact that matrix fragment peaks and sample fragment peaks do not coincide for several of low m/z fragments, hence we can identify sample fragment peaks relatively easy (i.e. 1037, 1021, 522, 258, 122, 80, etc)........ 12
Supplementary Figure 9. MALDI-LIFT-TOF/TOF MS^2 of MWLSA with HCCA matrix (bottom) and pure HCCA matrix (top) when collecting 1038.7090 m/z. The top spectrum was incorrectly named during collection, and should be named “20250509 HCCA PARENT 20+20 DA” since it is the parent spectrum collected for pure HCCA matrix, and is not a fragment spectrum. Y-axis display signal intensity, and x-axis display mass-over-charge (m/z) value. Most important note here is the fact that matrix fragment peaks and sample fragment peaks do not coincide for several of low m/z fragments, hence we can identify sample fragment peaks relatively easy (i.e. 965, 802, 759, 522, 258, 122, 80, etc). 13
Supplementary Figure 10. MALDI-TOF MS Spectra of HCCA saturated matrix used for MALDI-LIFT-TOF/TOF MS^2 analyses. Spectrum is collected in positive mode, range 0-5000 m/z, 300-1400 m/z region is displayed to give more clear view of registered peaks and due to no peaks being recorded outside of this region. Y-axis display signal intensity, and x-axis display mass-over-charge (m/z) value. 14
Supplementary Figure 11. ^{31}P-NMR spectra of unmodified and acetylated Spruce milled wood lignin (MWLSA). No hydroxyl signals are found in either aliphatic or phenolic region for MWLSA. Upper part is a ^{31}P-NMR from previous studies on Spruce MWL in our lab. Lower part is ^{31}P-NMR from this study. 15
Supplementary Figure 12. Explanation of derived m/z increments in MALDI-TOF MS analysis for uncondensed and condensed unmodified lignin bonds. 16
Supplementary Figure 13. Entire ^{13}C ^{1}H 2D-HSQC Spectrum of Spruce Milled Wood lignin (MWLS). Collected on a 900 MHz NMR spectrometer. Solvent used is DMSO-d_6. Temperature: 298.0 K........ 17
Supplementary Figure 14. Entire ^{13}C ^{1}H 2D-HSQC spectrum for synthetic lignin produced from HRP (ENZDHP). Collected on a 400 MHz NMR spectrometer. Solvent used is DMSO-d_6. Temperature: 298.0 K. 18
Supplementary Figure 15. Entire ^{13}C ^{1}H 2D-HSQC spectrum for synthetic lignin produced from $FeCl_3$. Collected on a 400 MHz NMR spectrometer. Solvent used is DMSO-d_6. Temperature: 298.0 K........ 19
Supplementary Figure 16. Entire ^{13}C ^{1}H 2D-HMBC spectrum for MWLS. Collected on a 400 MHz NMR spectrometer. Solvent used is DMSO-d_6. Temperature: 298.0 K........ 20
Supplementary Figure 17. MALDI-TOF MS spectra of acetylated kraft lignin from spruce. Samples were solubilized in DMF at 1.6 mg ml^{-1} concentration. Saturated HCCA in TA50 was used as matrix. Attempts were made to collect spectra with DHB as matrix, but failed. 0-5000 m/z was investigated. Y-axis display signal intensity, and x-axis display mass-over-charge (m/z) value. An expansion of this spectra is showcased in Supplementary Figure 18. 23
Supplementary Figure 18. Expansion of MALDI-TOF MS spectra of acetylated kraft lignin from spruce. Samples were solubilized in DMF at 1.6 mg ml^{-1} concentration. Saturated HCCA in TA50 was used as matrix. Attempts were made to collect spectra with DHB as matrix, but failed. 0-5000 m/z was investigated. Y-axis display signal intensity, and x-axis display mass-over-charge (m/z) value. Between some of the most intense peaks in the acetylated kraft sample a reoccurring increment of 88 can be found between 568 and 656 m/z, 612 and 700 m/z, 700 and 788 m/z, as well as 863 and 951 m/z. 24
Supplementary Figure 19. MALDI-TOF MS spectra of non-modified kraft lignin from spruce. Samples were solubilized in TA30 at 1 mg ml^{-1} concentration. DHB 20 mg ml^{-1} in TA30 was used as matrix. 0-5000 m/z was investigated. Y-axis display signal intensity, and x-axis display mass-over-charge (m/z) value. 25

Table of tables

Supplementary Table 1. Statistical analysis of m/z from inner crystallization spot used for creation of LPM obtained from MALDI-TOF MS. Inner and outer crystallization spots are showcased separately. In total, six replicates were measured (three example spectra representative of replicates are given in Supplementary Figure 6 and 7). 21
Supplementary Table 2. Statistical analysis of m/z from outer crystallization spot used for creation of LPM obtained from MALDI-TOF MS. Inner and outer crystallization spot are showcased separately. In total, six replicates were measured (three example spectra representative of replicates are given in Supplementary Figure 6 and 7). 22

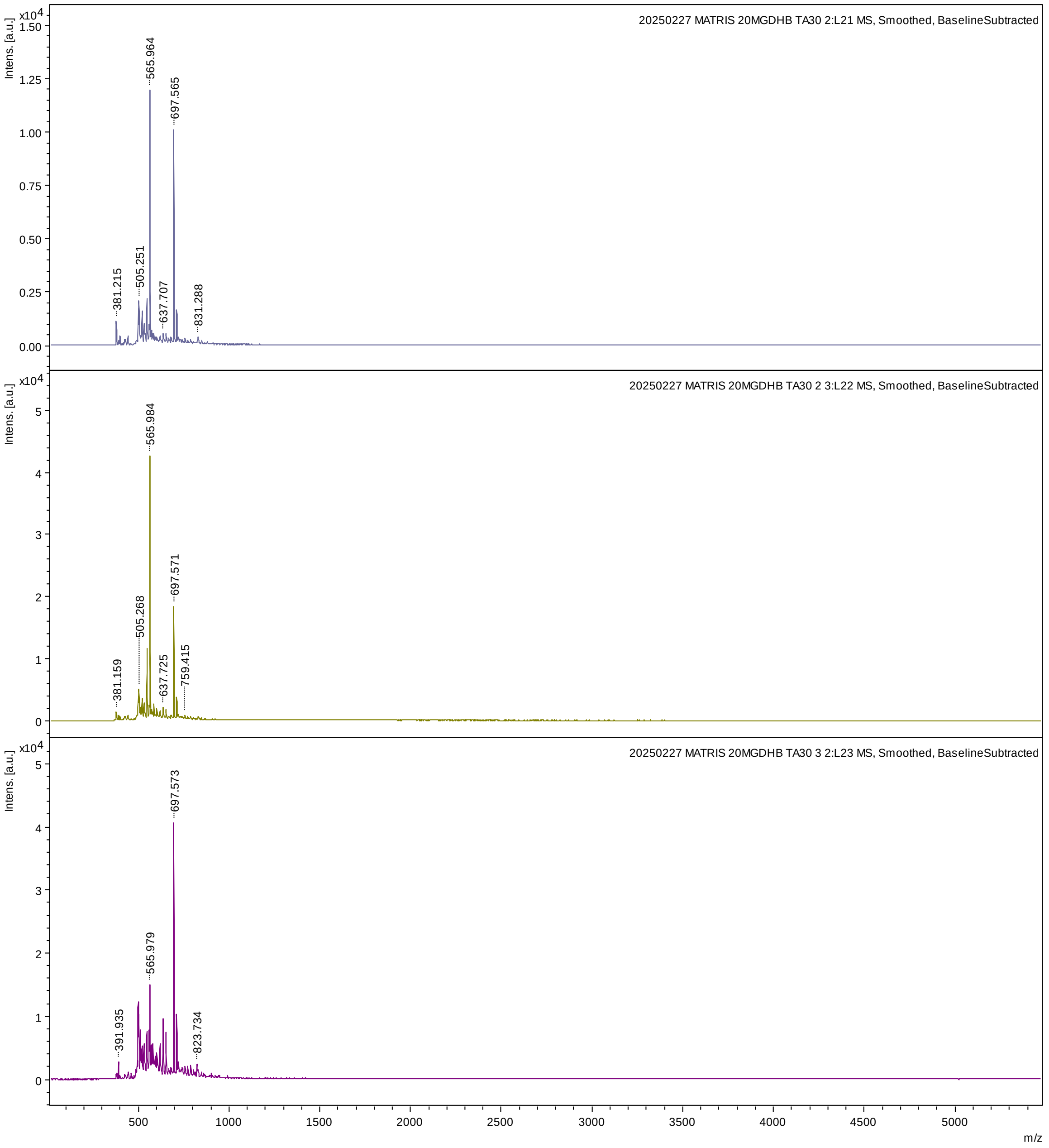

Supplementary Figure 1. MALDI-TOF spectra of pure 2,5-DHB matrix. *20 mg ml^{-1} in TA30+ 0.1% TFA. 0-5000 m/z was investigated. The peaks shown are those stemming from the pure matrix. Y-axis display signal intensity, and x-axis display mass-over-charge (m/z) value. Above approximately 831 m/z, no signal from the matrix is recorded.*

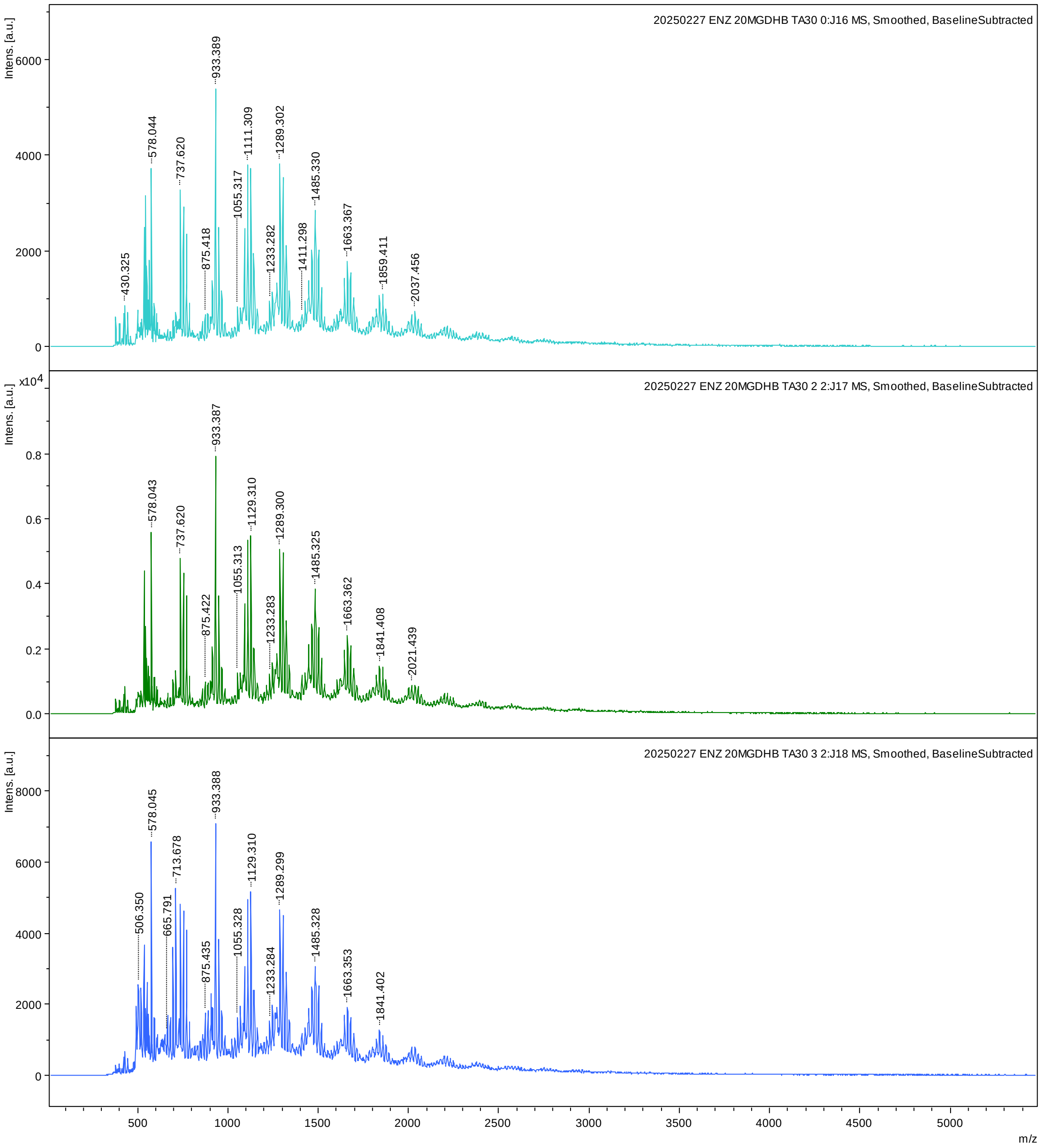

Supplementary Figure 2. MALDI-TOF MS spectra of ENZDHP. *0-5000 m/z was investigated. Y-axis display signal intensity, and x-axis display mass-over-charge (m/z) value. Clusters are representative of one monomer adding into the chain, which gives a spread in m/z given that the addition of a monomer with or without water and different connectivity yields different m/z increments. Weak clusters can be seen up to around 2500 m/z, but more intense clusters are seen until around 2000 m/z.*

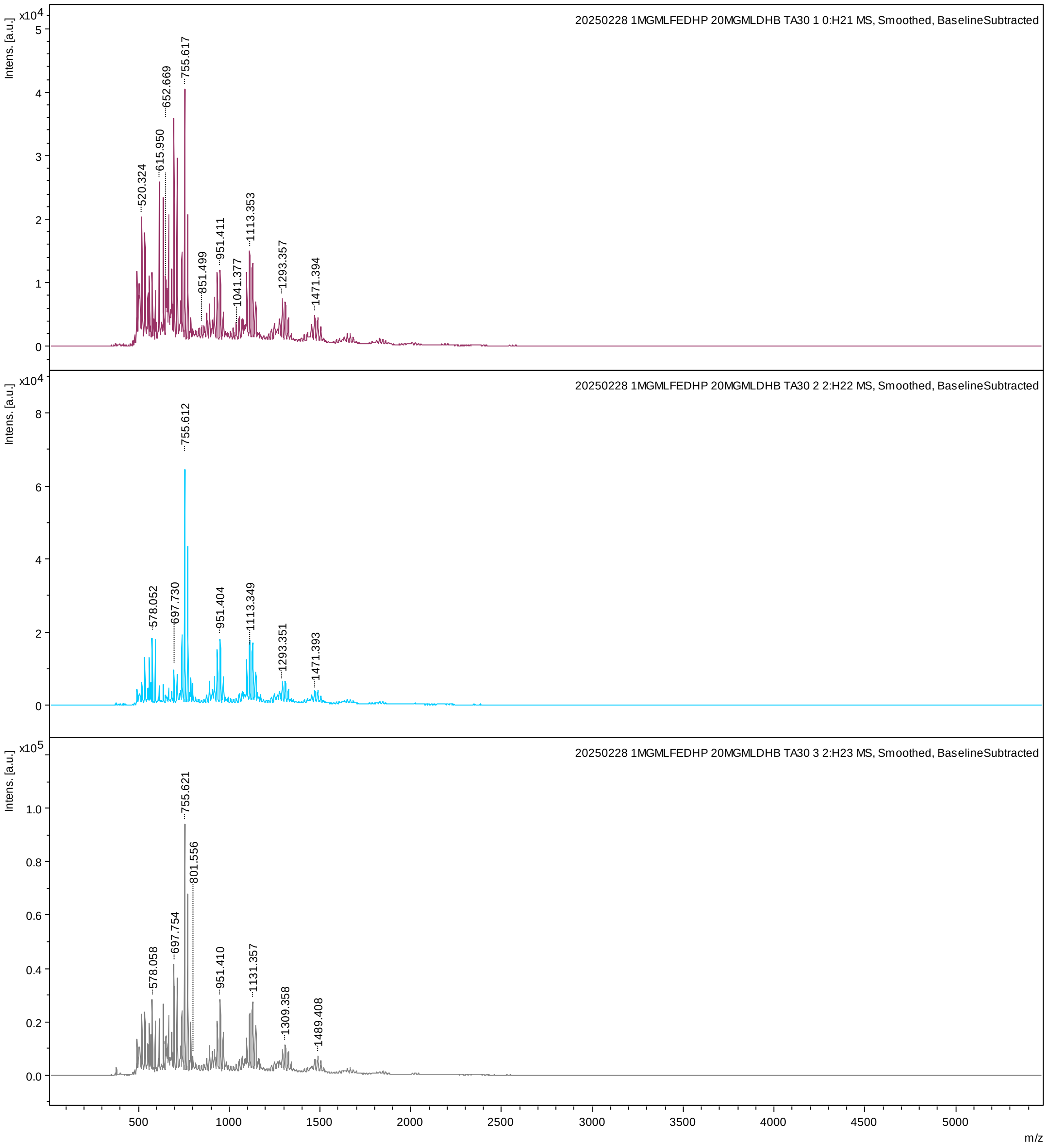

***Supplementary Figure 3. MALDI-TOF MS spectra of FEDHP**. 0-5000 m/z was investigated. Y-axis display signal intensity, and x-axis display mass-over-charge (m/z) value. Clusters are representative of one monomer adding into the chain, which gives a spread in m/z given that the addition of a monomer with or without water and different connectivity yields different m/z increments. Weak clusters are seen until approximately 2000 m/z, and stronger clusters are recorded up until around 1500 m/z.*

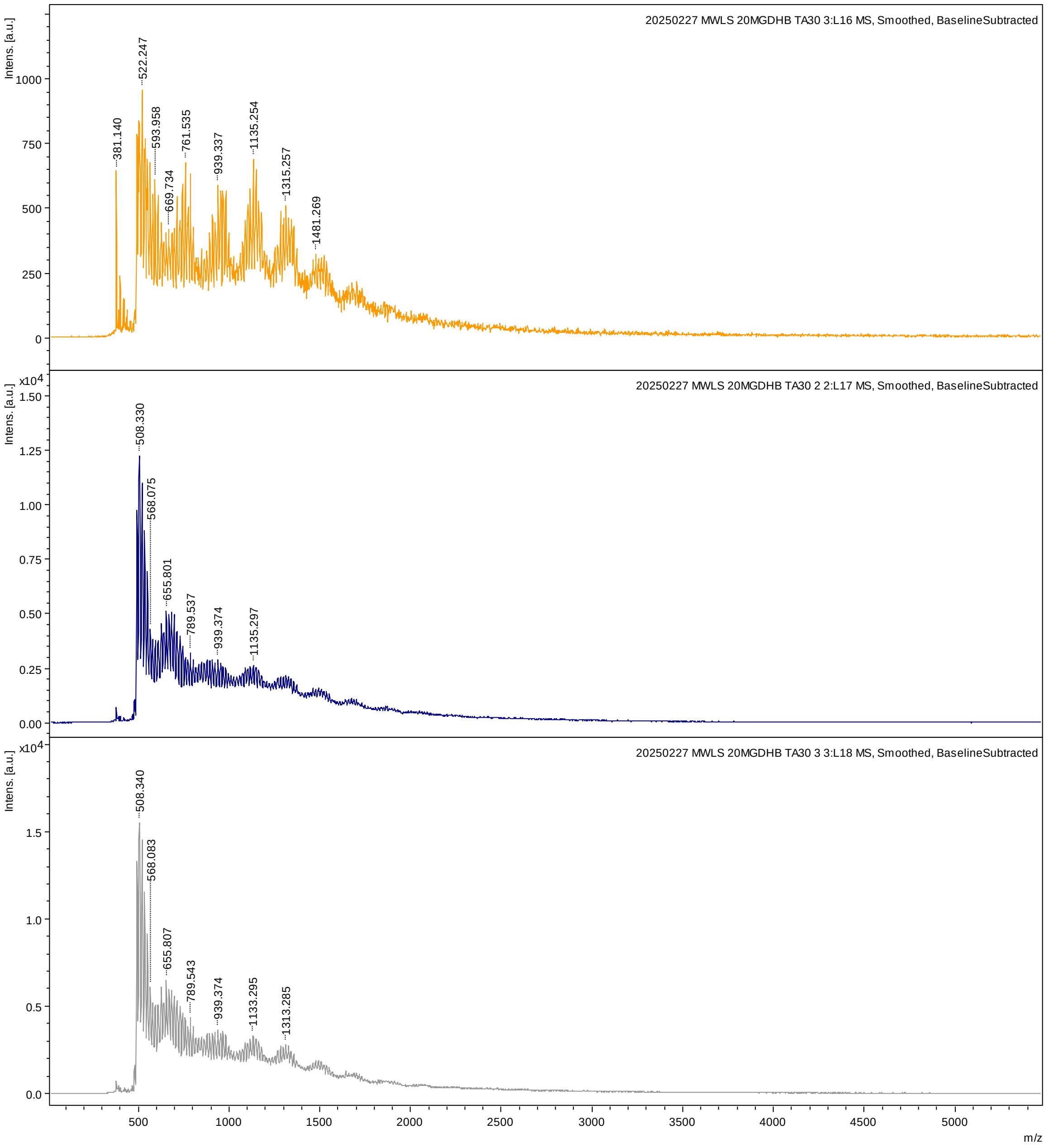

Supplementary Figure 4. MALDI-TOF MS spectra of MWLS. *0-5000 m/z was investigated. Y-axis display signal intensity, and x-axis display mass-over-charge (m/z) value. Clusters are representative of one monomer adding into the chain, which gives a spread in m/z given that the addition of a monomer with or without water and different connectivity yields different m/z increments. Clusters are seen up until 1700 m/z, with a broad slope tailing out around 2000 m/z.*

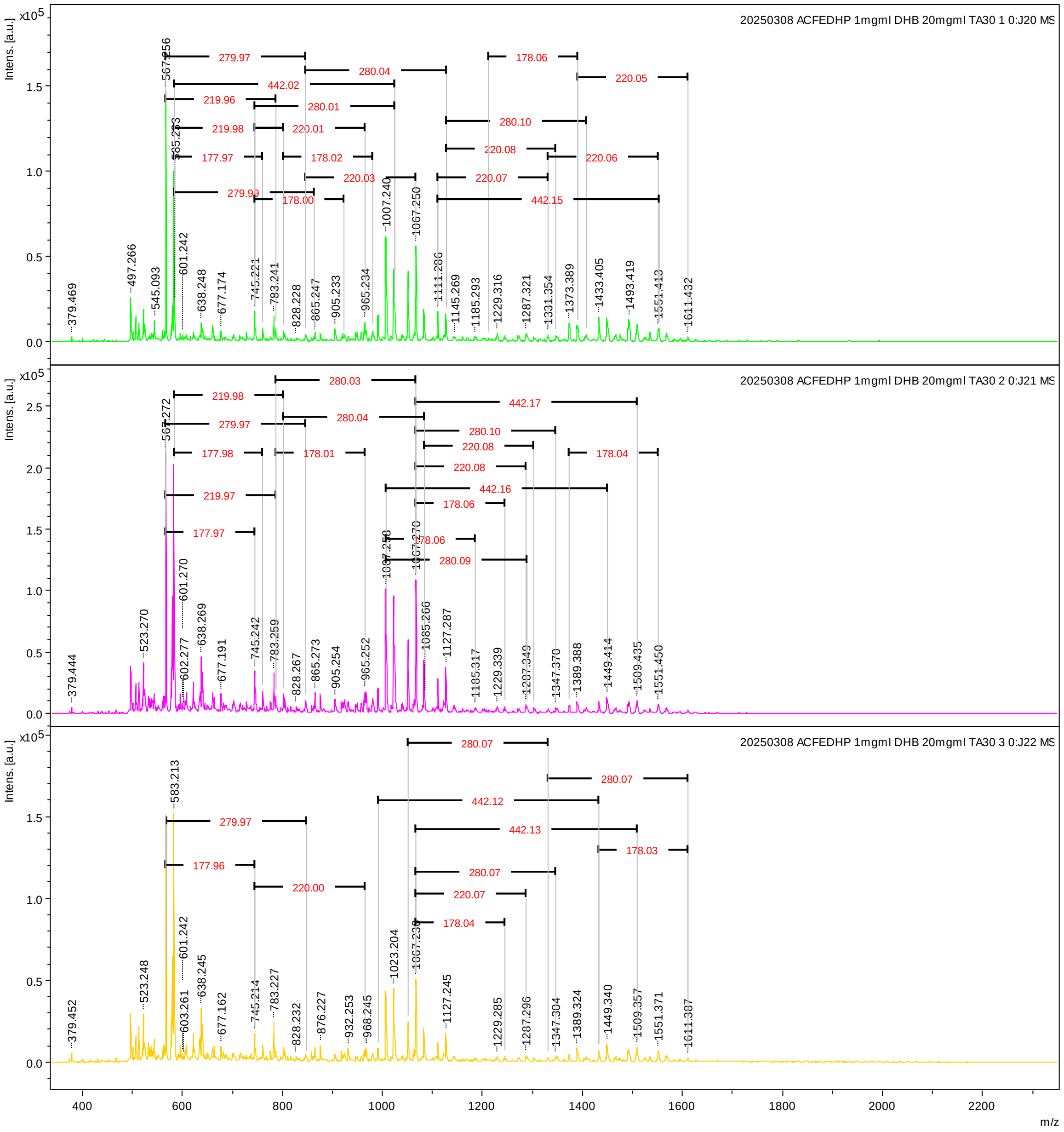

Supplementary Figure 5. MALDI-TOF MS spectra of acetylated FEDHP (ACFEDHP). *Spectra were acquired for 0-5000 m/z but to give a better overview of regions were acetylated m/z adducts are actually present, the spectra are zoomed in to 400-2200 m/z. Notice the m/z for acetylated β-O-4'-dimer (567 m/z) being among the most intense peaks in all spectra. Y-axis display signal intensity, and x-axis display mass-over-charge (m/z) value. Clusters are representative of one monomer adding into the chain, which gives a spread in m/z given that the addition of a monomer with or without water and different connectivity yields different m/z increments. Marked distances for the respective acetylated increments discussed in the article are marked, and 442 m/z is also found in the sample.*

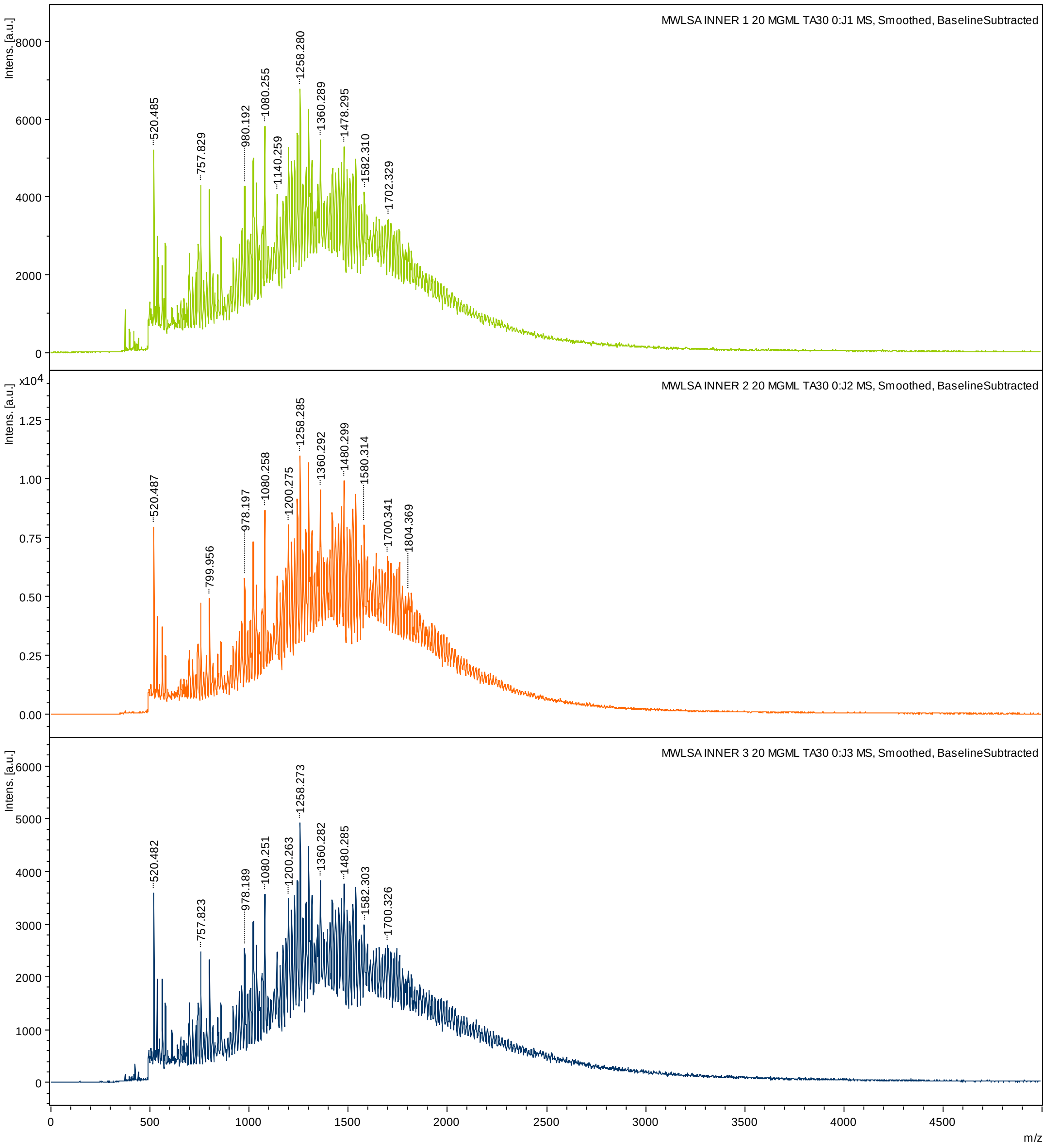

Supplementary Figure 6. MALDI-TOF MS spectra of acetylated MWLS (MWLSA) obtained from inner crystallization area on MALDI-TOF MS sample spots. *0-5000 m/z was investigated. Y-axis display signal intensity, and x-axis display mass-over-charge (m/z) value. Clusters are representative of one monomer adding into the chain, which gives a spread in m/z given that the addition of a monomer with or without water and different connectivity yields different m/z increments.*

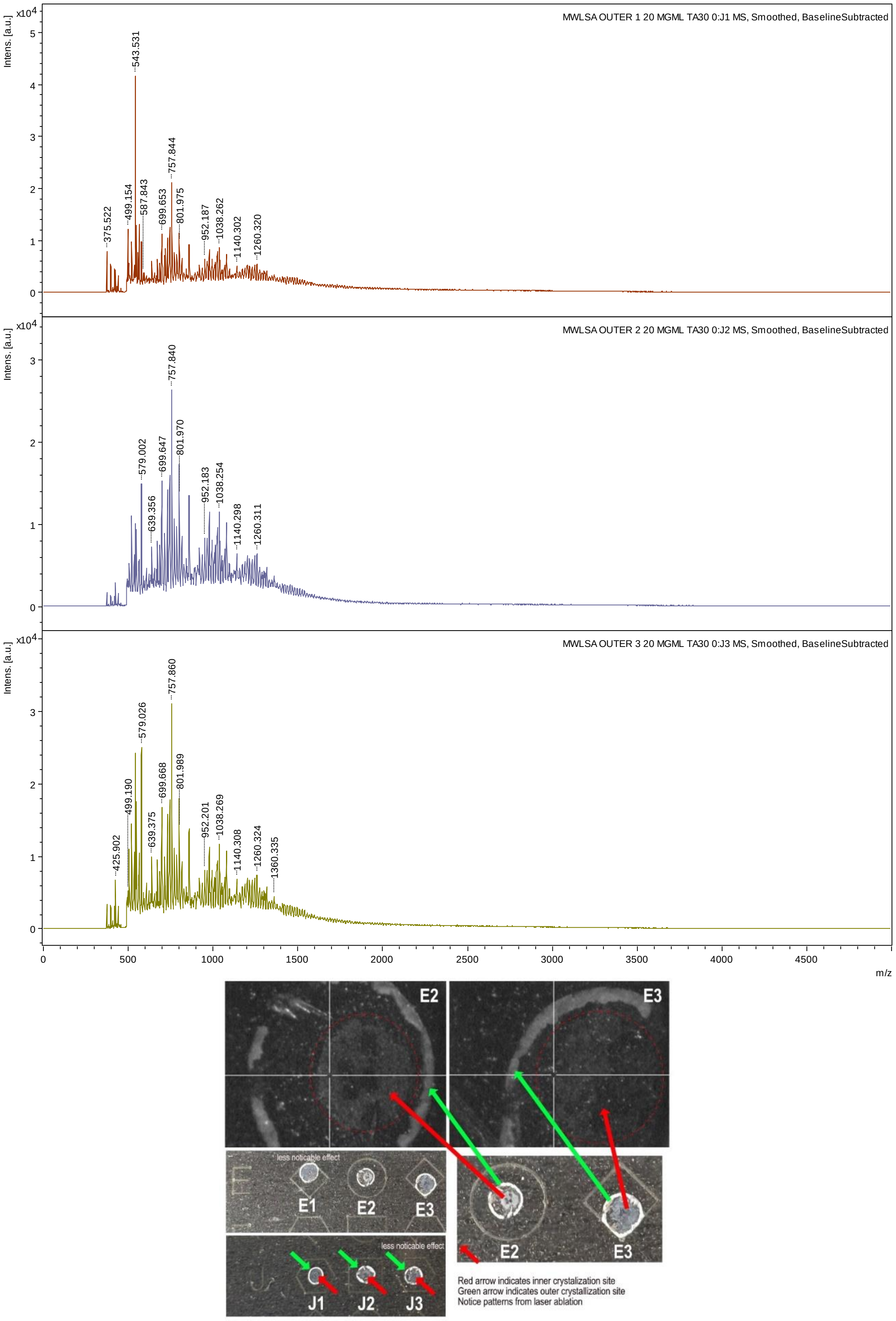

Supplementary Figure 7. MALDI-TOF MS spectra of acetylated MWLS (MWLSA) obtained from outer crystallization area on MALDI-TOF MS sample spots. *0-5000 m/z was investigated. Descriptive picture of crystallization regions is inserted underneath MALDI-TOF spectra. In spectra, Y-axis display signal intensity, and x-axis display mass-over-charge (m/z) value. Clusters are representative of one monomer adding into the chain, which gives a spread in m/z given that the addition of a monomer with or without water and different connectivity yields different m/z increments.*

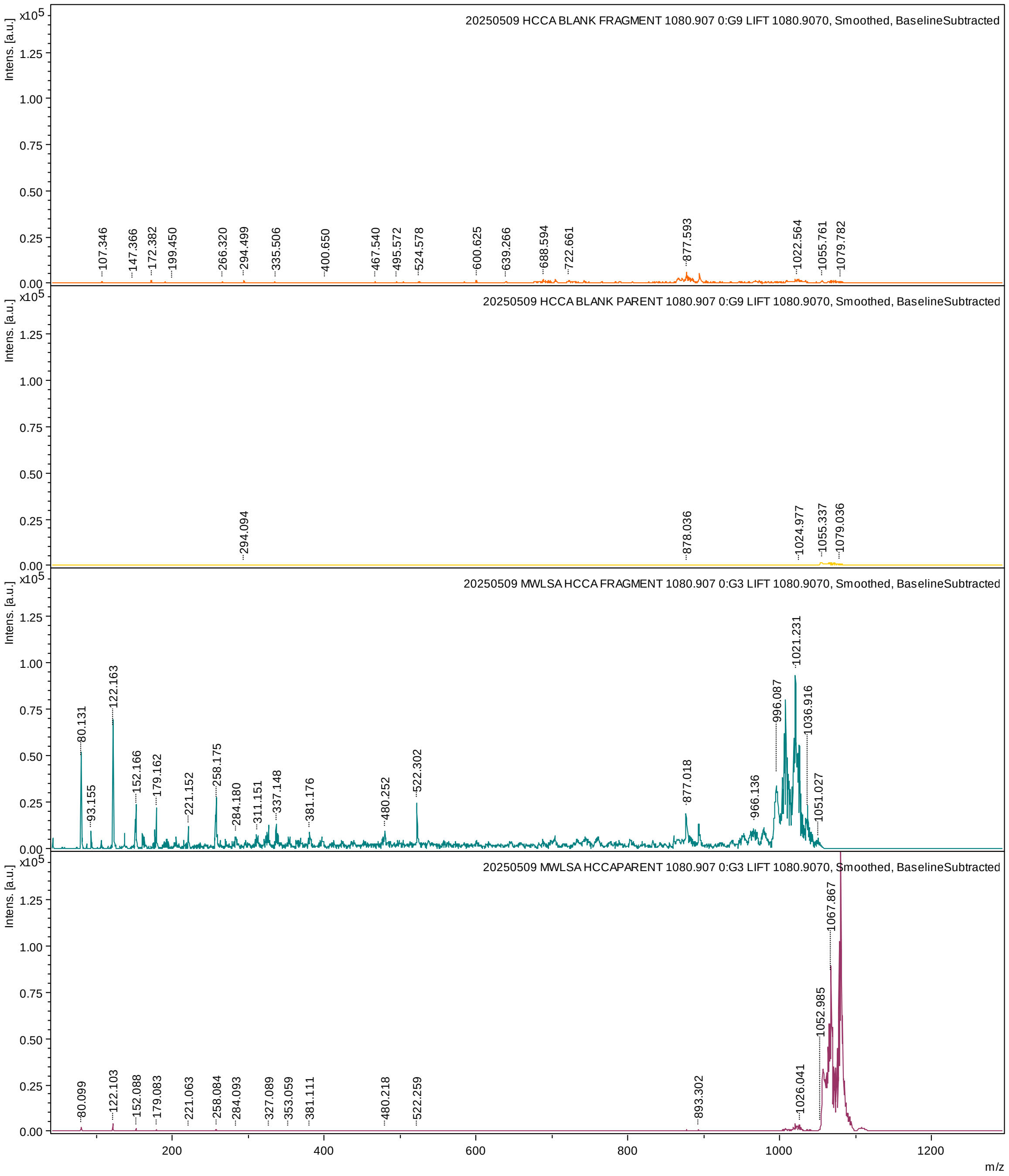

Supplementary Figure 8. MALDI-LIFT-TOF/TOF MS^2 of MWLSA with HCCA matrix (bottom) and pure HCCA matrix (top) when collecting 1080.9070 m/z. *Y-axis display signal intensity, and x-axis display mass-over-charge (m/z) value. Counting from top, 2nd and 4th spectra are parent spectra, while 1st and 3rd are fragment spectra. Most important note here is the fact that matrix fragment peaks and sample fragment peaks do not coincide for several of low m/z fragments, hence we can identify sample fragment peaks relatively easy (i.e. 1037, 1021, 522, 258, 122, 80, etc).*

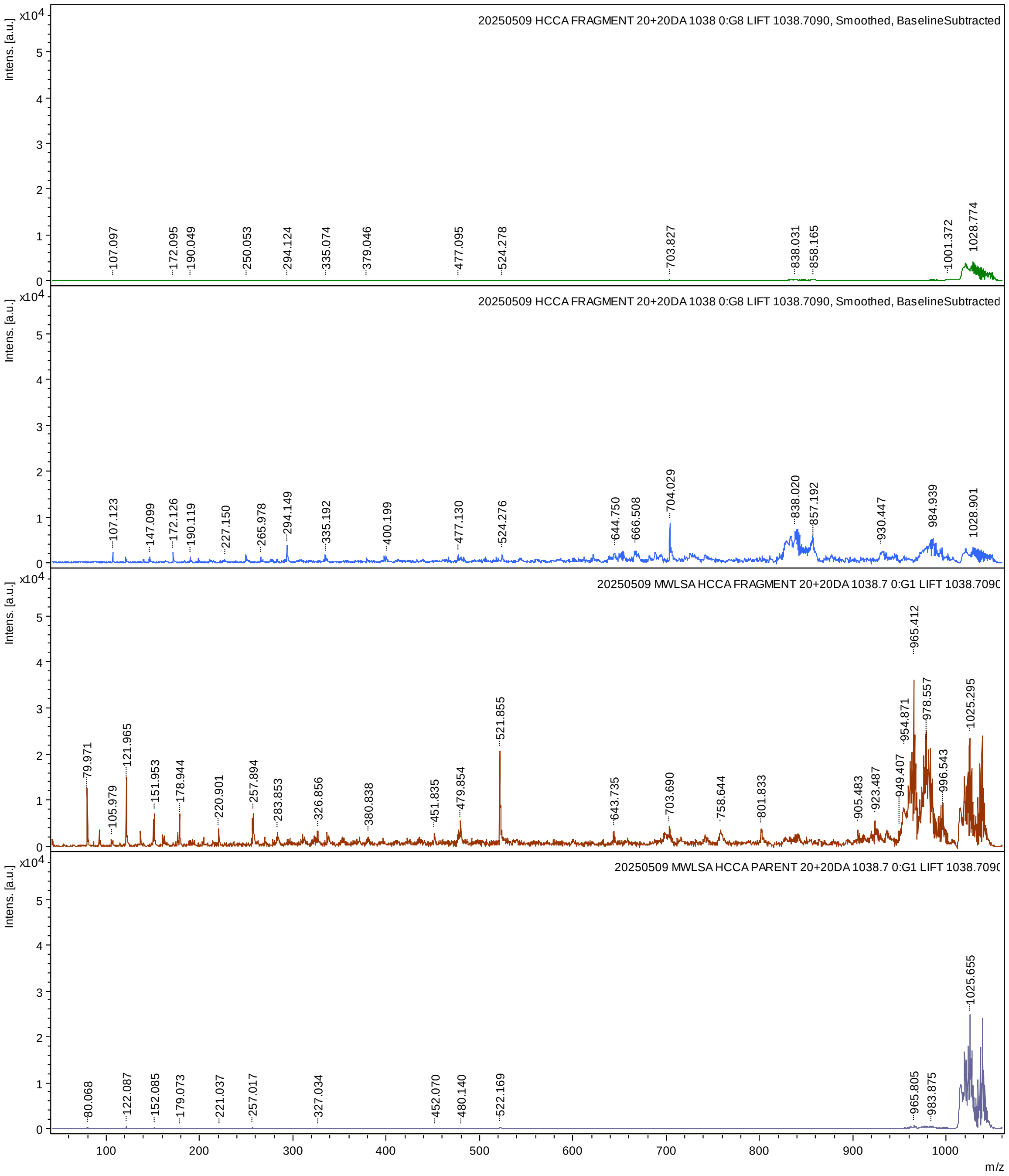

Supplementary Figure 9. MALDI-LIFT-TOF/TOF MS^2 of MWLSA with HCCA matrix (bottom) and pure HCCA matrix (top) when collecting 1038.7090 m/z. *The <u>top spectrum was incorrectly named during collection</u>, and should be named "20250509 HCCA PARENT 20+20 DA" since it is the parent spectrum collected for pure HCCA matrix, and is not a fragment spectrum. Y-axis display signal intensity, and x-axis display mass-over-charge (m/z) value. Most important note here is the fact that matrix fragment peaks and sample fragment peaks do not coincide for several of low m/z fragments, hence we can identify sample fragment peaks relatively easy (i.e. 965, 802, 759, 522, 258, 122, 80, etc).*

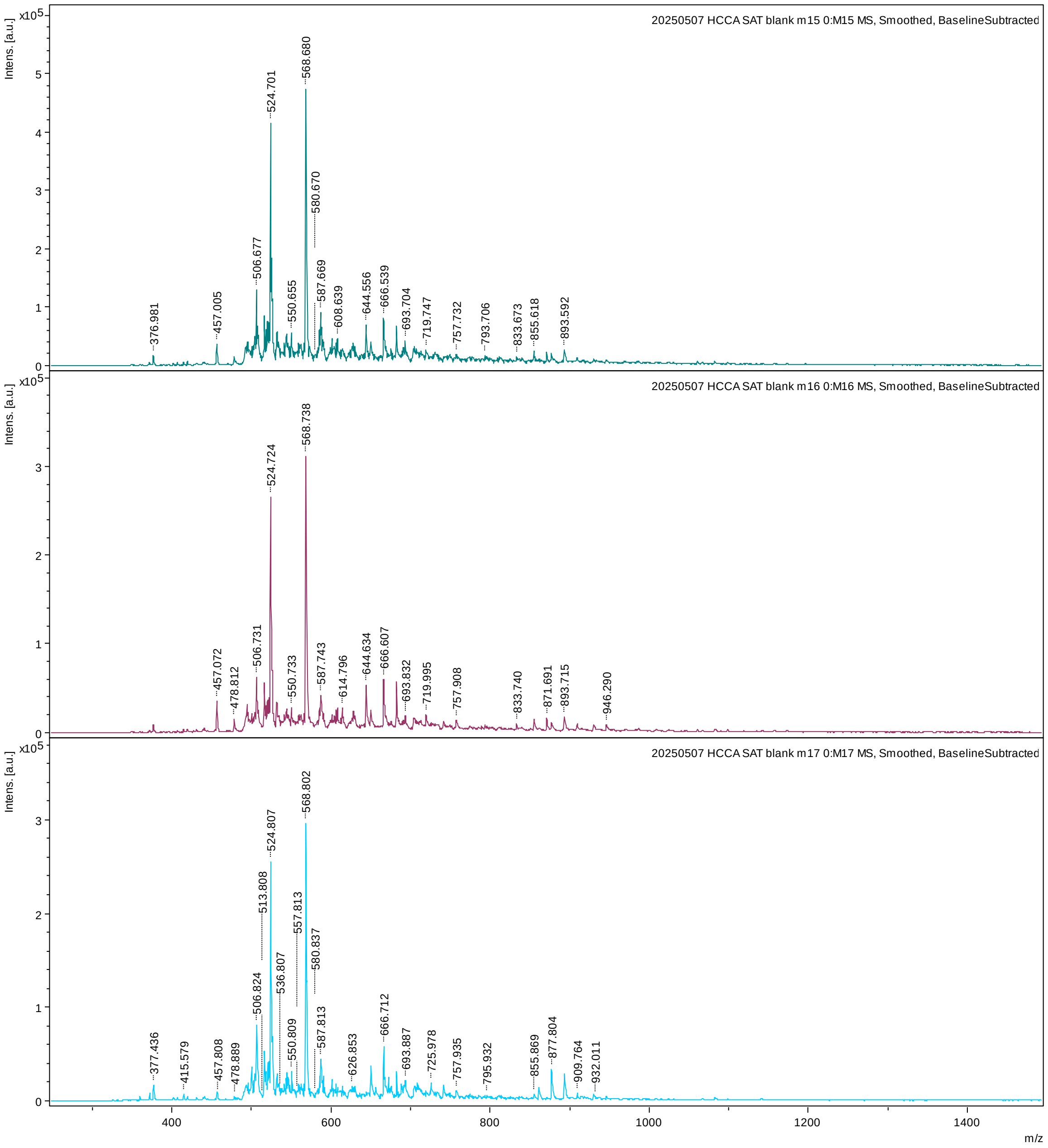

Supplementary Figure 10. MALDI-TOF MS Spectra of HCCA saturated matrix used for MALDI-LIFT-TOF/TOF MS^2 analyses. *Spectrum is collected in positive mode, range 0-5000 m/z, 300-1400 m/z region is displayed to give more clear view of registered peaks and due to no peaks being recorded outside of this region. Y-axis display signal intensity, and x-axis display mass-over-charge (m/z) value.*

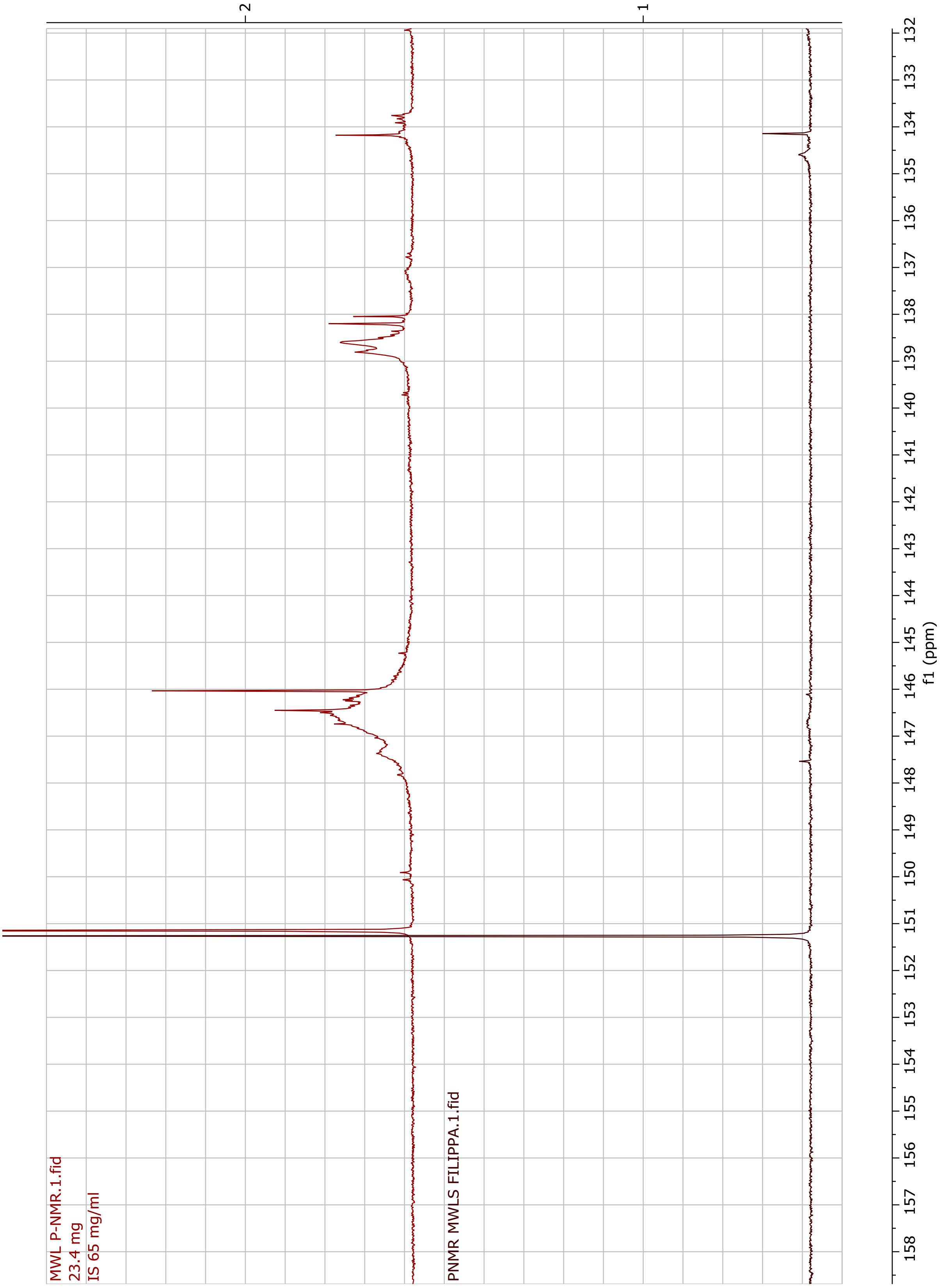

Supplementary Figure 11. ^{31}P-NMR spectra of unmodified and acetylated Spruce milled wood lignin (MWLSA). *No hydroxyl signals are found in either aliphatic or phenolic region for MWLSA. Upper part is a ^{31}P-NMR from previous studies on Spruce MWL in our lab. Lower part is ^{31}P-NMR from this study.*

Fingerprinting mass increments of 196 and 178 m/z

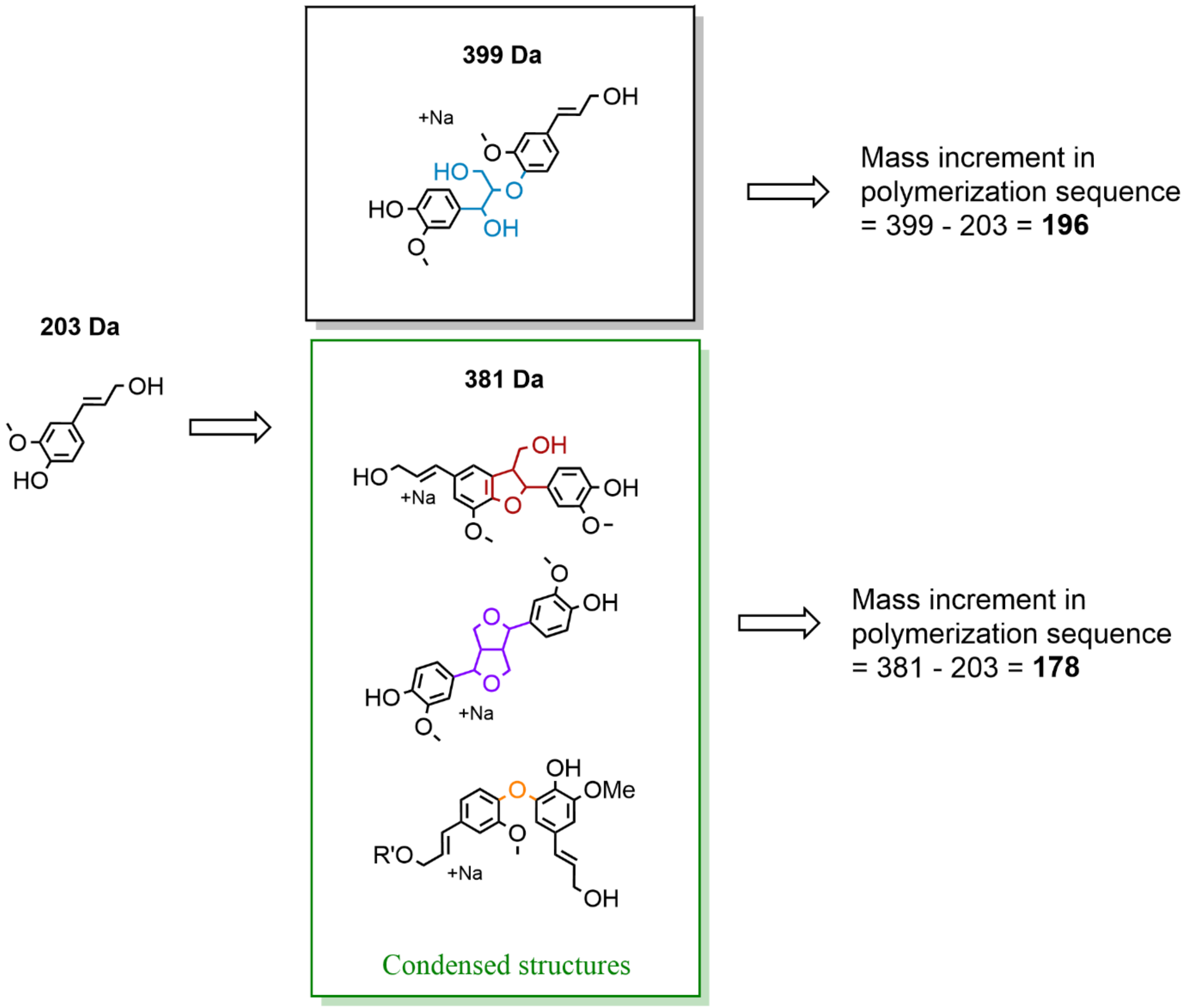

Supplementary Figure 12. Explanation of derived m/z increments in MALDI-TOF MS analysis for uncondensed and condensed unmodified lignin bonds.

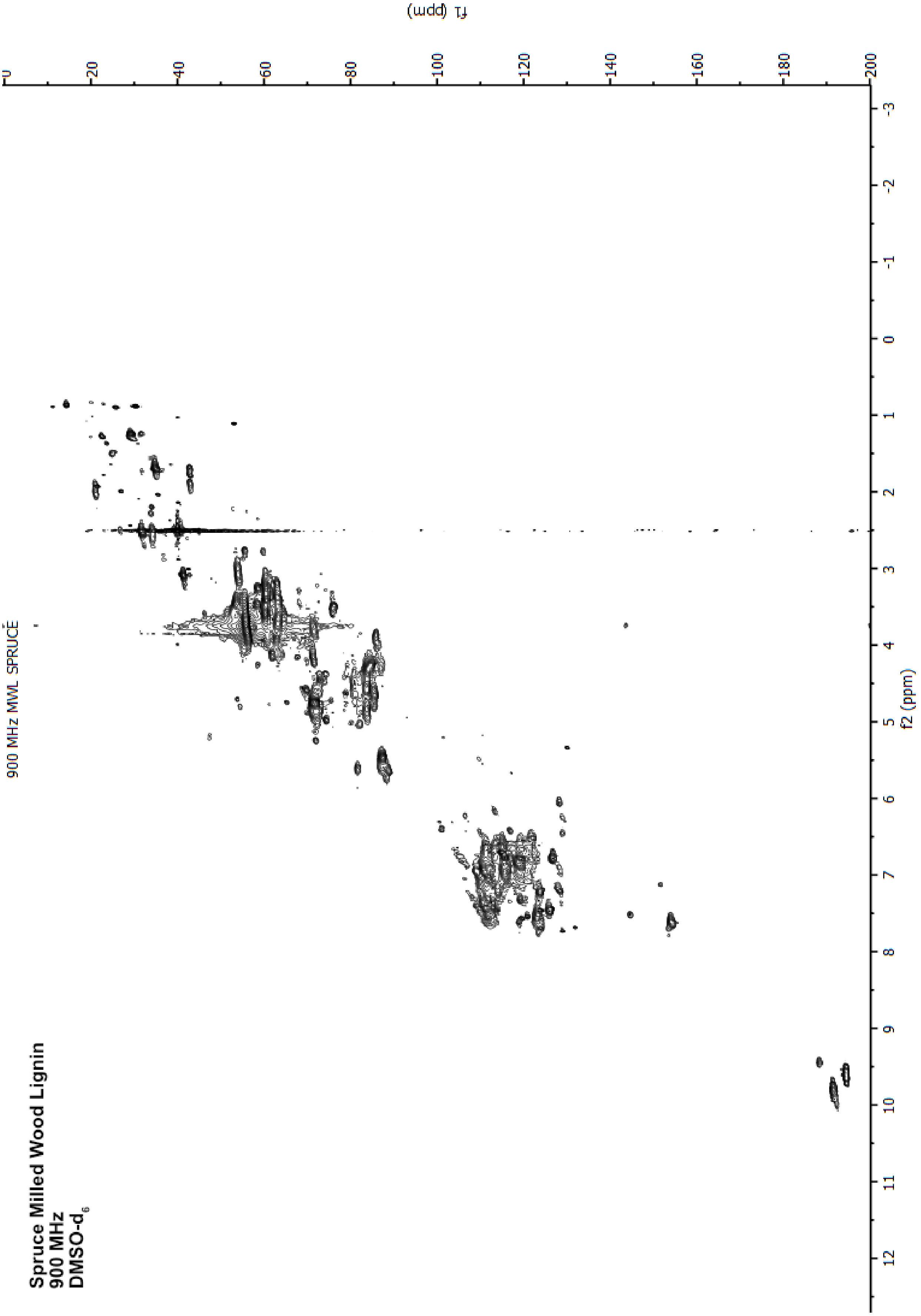

Supplementary Figure 13. Entire ^{13}C ^{1}H 2D-HSQC Spectrum of Spruce Milled Wood lignin (MWLS).
Collected on a 900 MHz NMR spectrometer. Solvent used is DMSO-d$_6$. Temperature: 298.0 K.

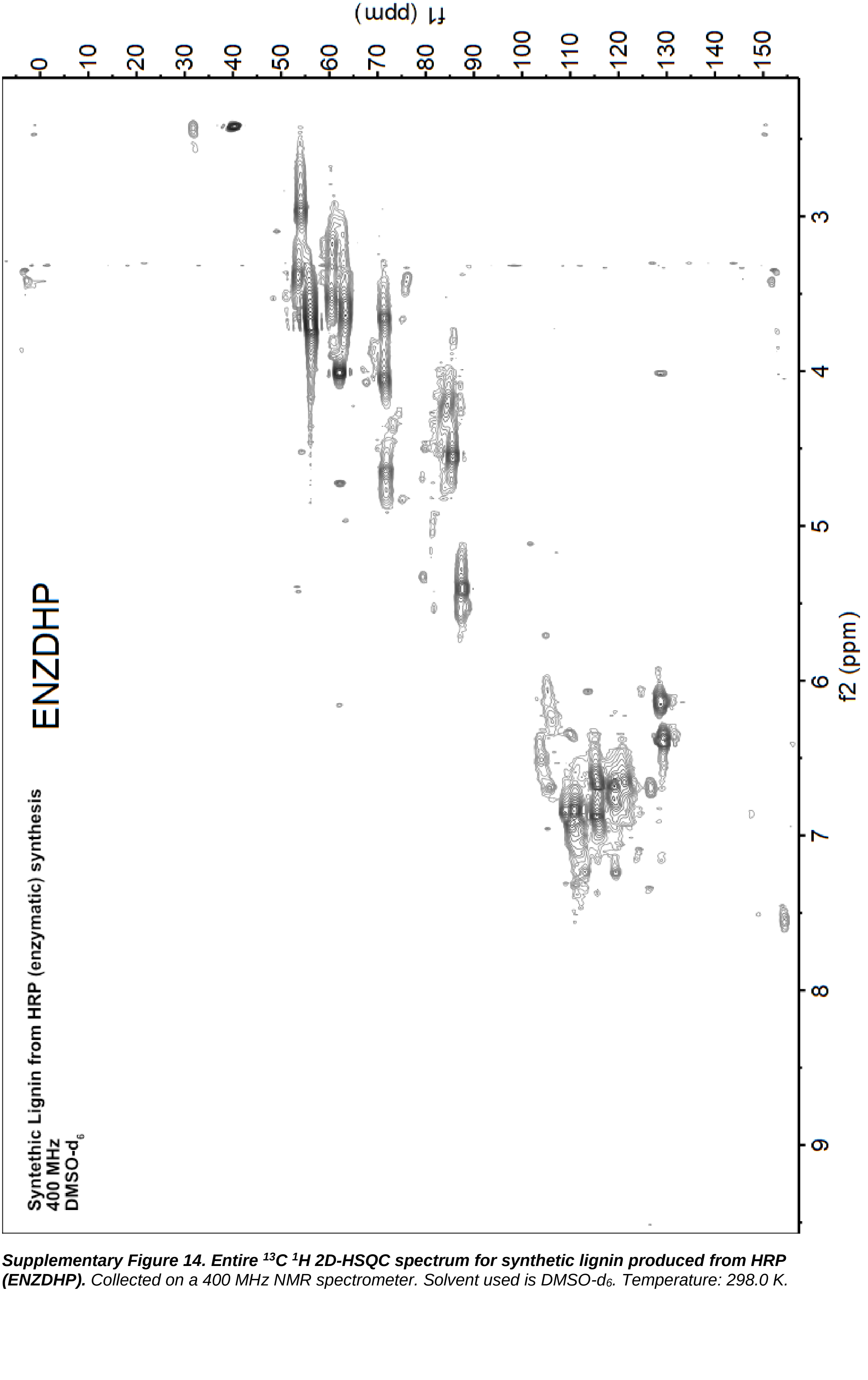

Supplementary Figure 14. Entire ^{13}C ^{1}H 2D-HSQC spectrum for synthetic lignin produced from HRP (ENZDHP). *Collected on a 400 MHz NMR spectrometer. Solvent used is DMSO-d_6. Temperature: 298.0 K.*

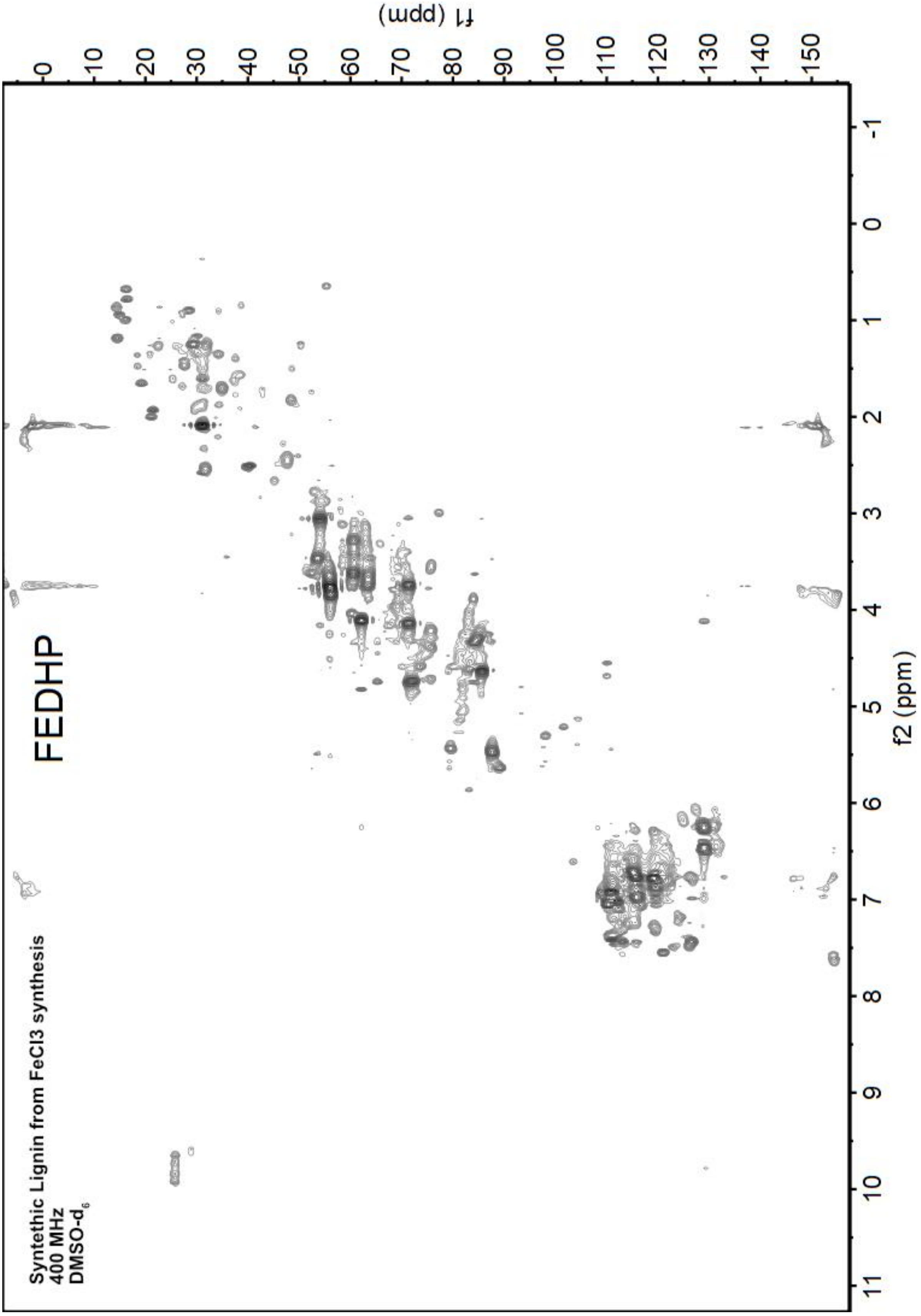

Supplementary Figure 15. Entire ^{13}C ^{1}H 2D-HSQC spectrum for synthetic lignin produced from $FeCl_3$. *Collected on a 400 MHz NMR spectrometer. Solvent used is DMSO-d_6. Temperature: 298.0 K.*

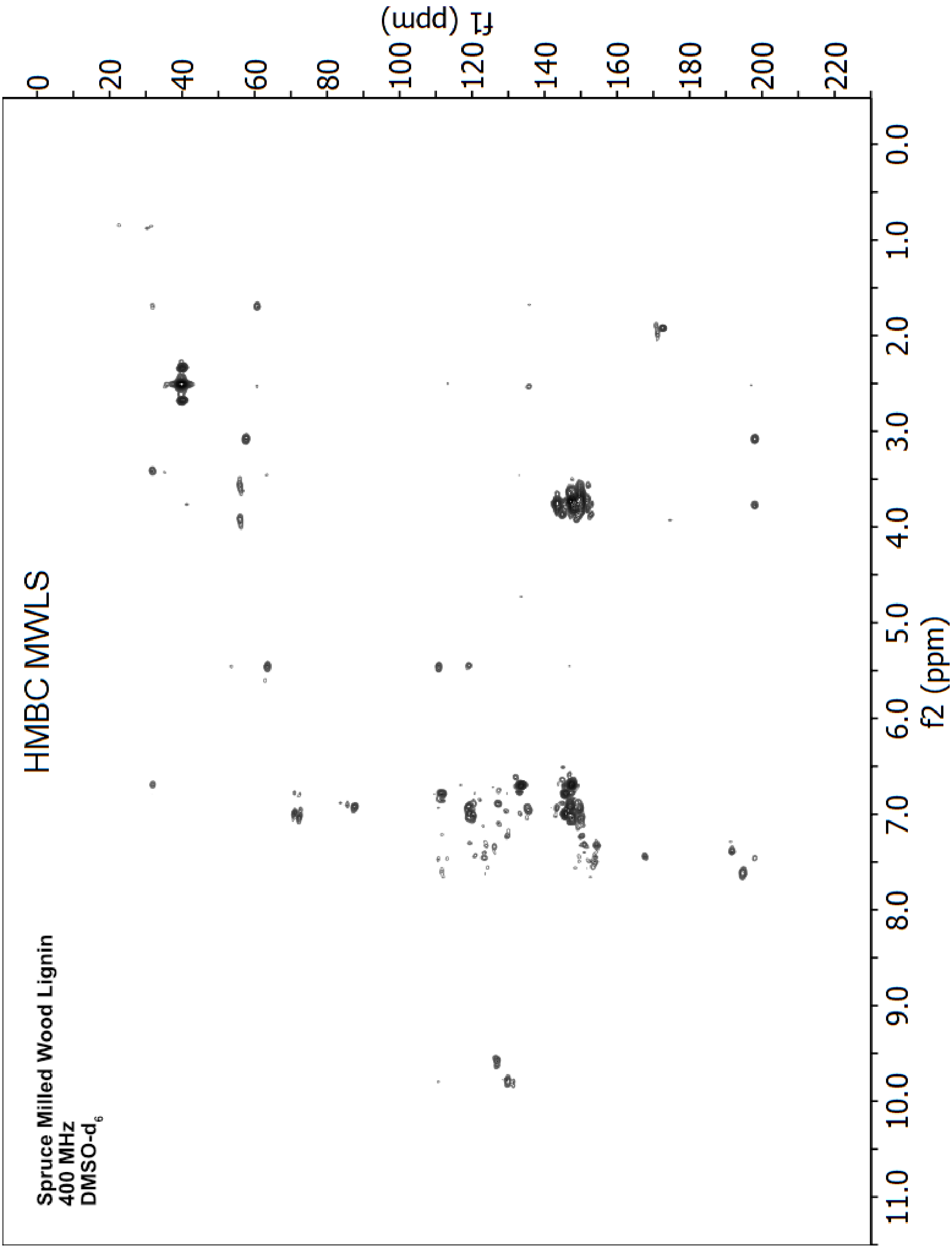

Supplementary Figure 16. Entire ^{13}C ^{1}H 2D-HMBC spectrum for MWLS. *Collected on a 400 MHz NMR spectrometer. Solvent used is DMSO-d_6. Temperature: 298.0 K.*

Supplementary Table 1. Statistical analysis of m/z from inner crystallization spot used for creation of LPM obtained from MALDI-TOF MS. *Inner and outer crystallization spots are showcased separately. In total, six replicates were measured (three example spectra representative of replicates are given in Supplementary Figure 6 and 7).*

INNER CRYSTALLIZATION SITE MWLSA							
**LPM [m/z]**	**No. of replicates where the peak was detected**	**S/N [a.u]**	**S/N sd [a.u]**	**S/N rsd [%]**	**m/z [Da]**	**m/z sd [Da]** x 10^{-3}	**m/z rsd [%]** x 10^{-3}
**520**	6	72.64	21.53	29.64	520.49	5.13	0.98
**740**	0	N/A	N/A	N/A	N/A	N/A	N/A
**800**	6	33.10	8.44	25.51	799.96	6.54	0.82
**918**	0	N/A	N/A	N/A	N/A	N/A	N/A
**1020**	6	33.81	7.84	23.19	1020.23	6.99	0.68
**1080**	6	38.08	8.88	23.33	1080.26	5.88	0.54
**1138**	0	N/A	N/A	N/A	N/A	N/A	N/A
**1198**	6	28.58	7.74	27.10	1198.28	7.48	0.62
**1240**	6	22.00	5.51	25.10	1240.28	5.57	0.45
**1300**	6	34.89	9.51	27.26	1300.29	5.99	0.46
**1316**	6	24.41	5.93	24.31	1316.28	5.01	0.38
**1360**	6	28.88	7.83	27.10	1360.29	6.69	0.49
**1418**	6	20.22	5.64	27.90	1418.29	7.27	0.51
**1478**	6	25.96	8.85	34.10	1478.30	7.46	0.50
**1520**	5	19.78	4.96	25.06	1520.30	11.66	0.77
**1580**	6	19.77	6.63	33.55	1580.31	5.83	0.37
**1638**	3	13.21	3.15	23.84	1638.31	11.37	0.69
**1640**	6	17.03	5.60	32.88	1640.33	7.00	0.43
**1698**	3	13.83	3.59	25.95	1698.33	12.10	0.71
**1758**	5	16.32	4.33	26.50	1758.35	8.05	0.46

For statistical analysis, six replicates of MWLSA (acetylated MWL from spruce) were run. *“No. of replicates where the peak was detected”* dictate how many times this specific peak was detected within the samples. Every reported value is reported as a mean value for the found peaks (i.e. if a peak is found “6” times, the mean is an average of 6 values). Since absolute peak intensity varies between spectra, we have chosen to report S/N instead.

Mean values are calculated using built-in function *‘average’* in Excel.

Standard deviation (m/z sd [Da]) is calculated using built-in function *‘stdev.s’* in Excel.

Relative standard deviation is further calculated by dividing the standard deviation with the mean value and converting into percentage according to:

$$rsd\ [\%] = \frac{\frac{m}{z} sd\ [Da]}{\frac{m}{z} [Da]} \times 100\%$$

Supplementary Table 2. Statistical analysis of m/z from outer crystallization spot used for creation of LPM obtained from MALDI-TOF MS. *Inner and outer crystallization spot are showcased separately. In total, six replicates were measured (three example spectra representative of replicates are given in Supplementary Figure 6 and 7).*

**OUTER CRYSTALLIZATION SITE MWLSA**							
**LPM [m/z]**	**No. of replicates where the peak was detected**	**S/N [a.u]**	**S/N sd [a.u]**	**S/N rsd [%]**	**m/z [Da]**	**m/z sd [Da]** x 10^{-3}	**rsd [%]** x 10^{-3}
**520**	2	20.55	4.12	20.27	519.92	572.05	110.03
**740**	6	21.55	9.45	43.86	739.81	6.48	0.88
**800**	6	54.69	25.27	46.20	799.98	7.00	0.88
**918**	4	17.99	6.78	37.69	918.16	6.18	0.67
**1020**	6	25.41	11.99	47.21	1020.26	6.71	0.66
**1080**	6	35.02	17.35	49.54	1080.29	6.11	0.57
**1138**	5	18.74	6.83	36.47	1138.29	4.62	0.41
**1198**	5	20.60	6.88	33.40	1198.31	4.74	0.40
**1240**	0	N/A	N/A	N/A	N/A	N/A	N/A
**1300**	5	20.92	7.07	33.81	1300.31	41.79	3.21
**1316**	3	18.05	6.26	34.71	1316.32	3.46	0.26
**1360**	3	18.07	6.50	36.00	1360.33	2.52	0.19
**1418**	0	N/A	N/A	N/A	N/A	N/A	N/A
**1478**	0	N/A	N/A	N/A	N/A	N/A	N/A
**1520**	0	N/A	N/A	N/A	N/A	N/A	N/A
**1580**	0	N/A	N/A	N/A	N/A	N/A	N/A
**1638**	0	N/A	N/A	N/A	N/A	N/A	N/A
**1640**	0	N/A	N/A	N/A	N/A	N/A	N/A
**1698**	0	N/A	N/A	N/A	N/A	N/A	N/A
**1758**	0	N/A	N/A	N/A	N/A	N/A	N/A

For statistical analysis, six replicates of MWLSA (acetylated MWL from spruce) were run. *"No. of replicates where the peak was detected"* dictate how many times this specific peak occurred within the samples. Every reported value is reported as a mean value for the found peaks (i.e. if a peak is found "6" times, the mean values calculated is an average of 6 values). Since absolute peak intensity varies between spectra, we have chosen to report S/N instead.

Mean values are calculated using built-in function *'average'* in Excel.

Standard deviation (m/z sd [Da]) is calculated using built-in function *'stdev.s'* in Excel.

Relative standard deviation is further calculated by dividing the standard deviation with the mean value and converting into percentage according to:

$$rsd\ [\%] = \frac{\frac{m}{z}\ sd\ [Da]}{\frac{m}{z}\ [Da]} \times 100\%$$

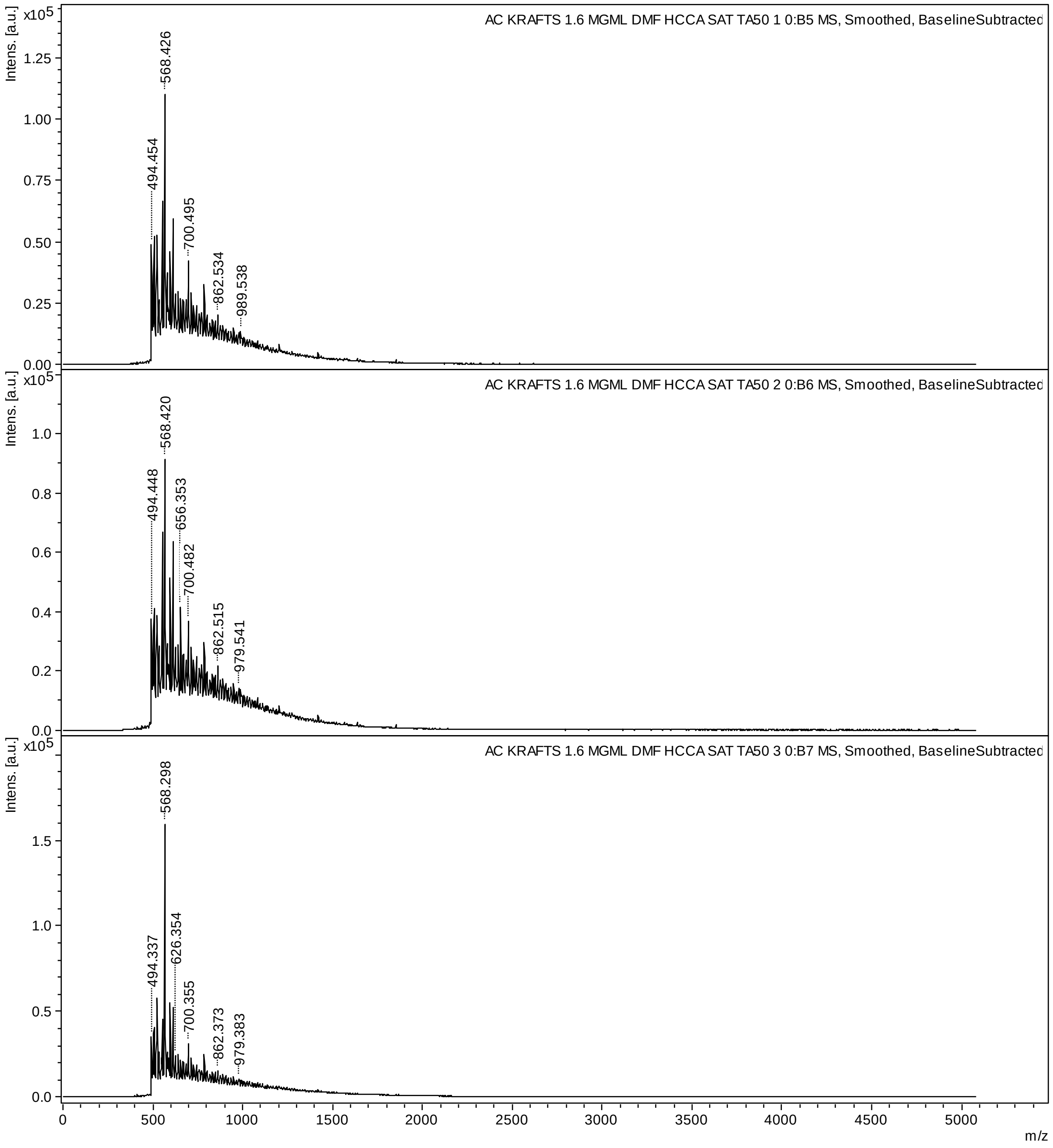

Supplementary Figure 17. MALDI-TOF MS spectra of acetylated kraft lignin from spruce. *Samples were solubilized in DMF at 1.6 mg ml^{-1} concentration. Saturated HCCA in TA50 was used as matrix. Attempts were made to collect spectra with DHB as matrix, but failed. 0-5000 m/z was investigated. Y-axis display signal intensity, and x-axis display mass-over-charge (m/z) value. An expansion of this spectra is showcased in Supplementary Figure 18.*

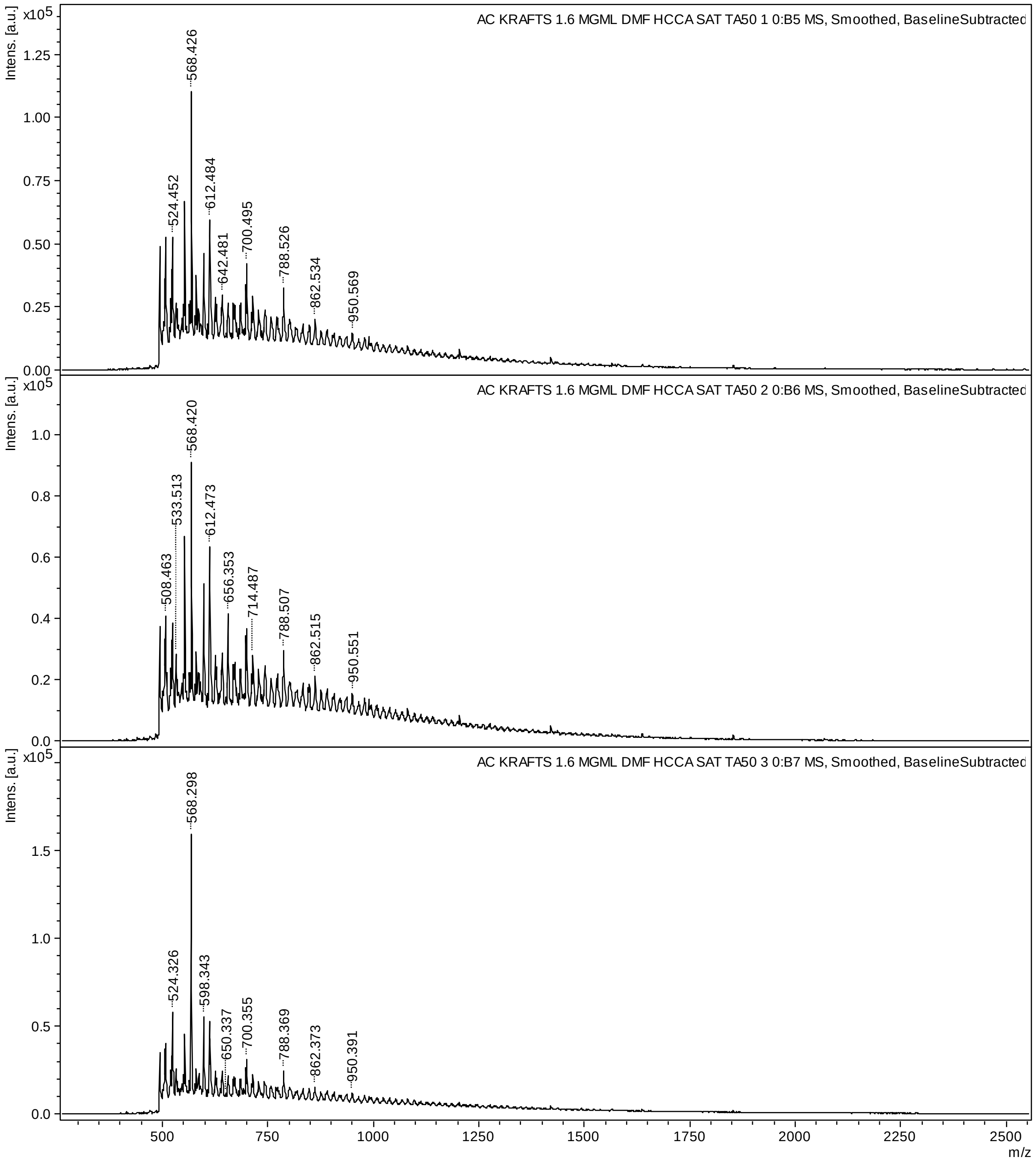

Supplementary Figure 18. Expansion of MALDI-TOF MS spectra of acetylated kraft lignin from spruce. *Samples were solubilized in DMF at 1.6 mg ml^{-1} concentration. Saturated HCCA in TA50 was used as matrix. Attempts were made to collect spectra with DHB as matrix, but failed. 0-5000 m/z was investigated. Y-axis display signal intensity, and x-axis display mass-over-charge (m/z) value. Between some of the most intense peaks in the acetylated kraft sample a reoccurring increment of 88 can be found between 568 and 656 m/z, 612 and 700 m/z, 700 and 788 m/z, as well as 863 and 951 m/z.*

When compared to the acetylated MWL from spruce, there is no clear repeat unit or polymerization clusters in the spectra that are similar (i.e. the 280, 220, and 178 m/z increments). However, between some of the most intense peaks in the acetylated kraft sample, a reoccurring increment of 88 or 176 can be found but we have not been able to postulate a structure for this specific increment.

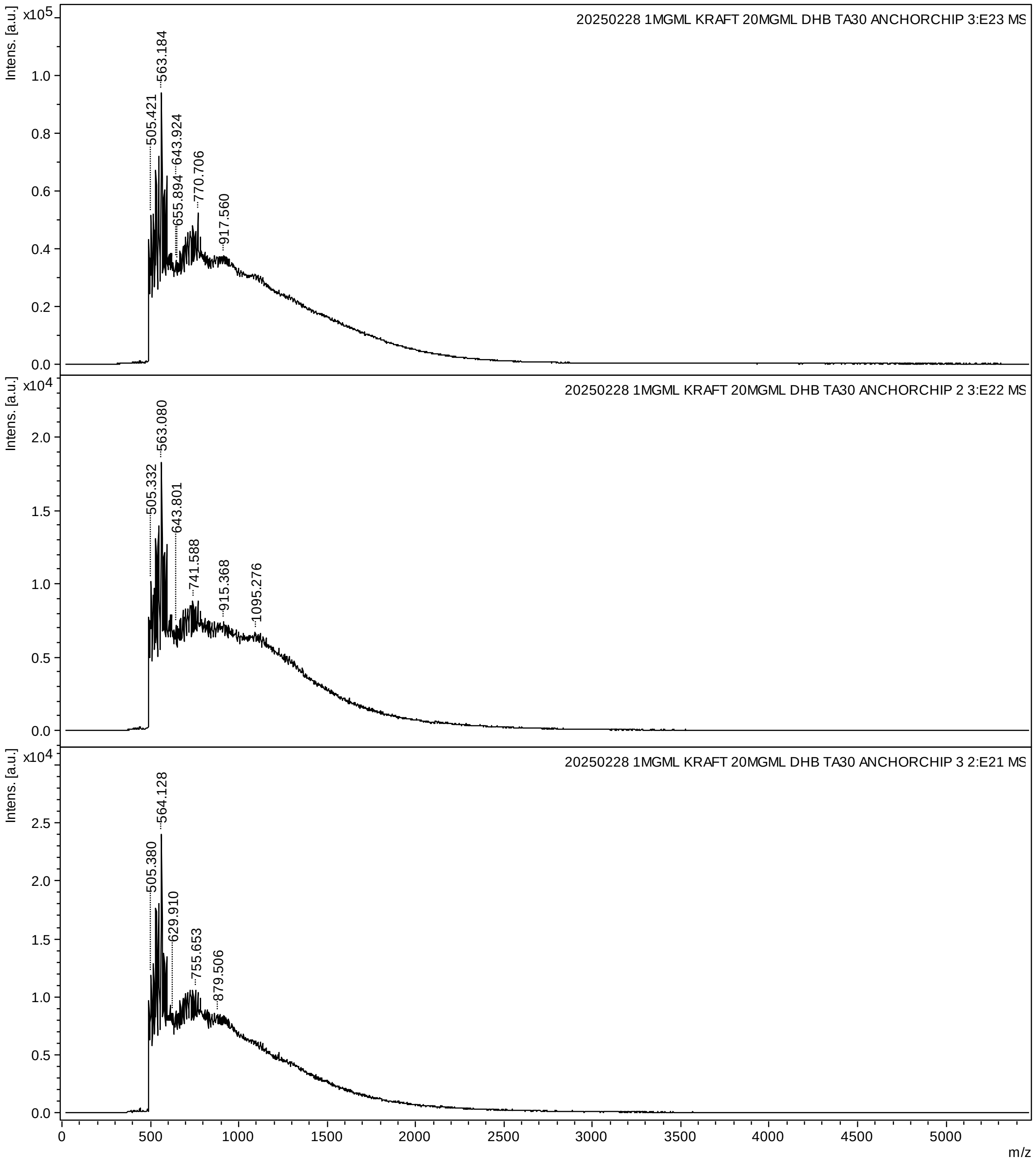

Supplementary Figure 19. MALDI-TOF MS spectra of non-modified kraft lignin from spruce. *Samples were solubilized in TA30 at 1 mg ml^{-1} concentration. DHB 20 mg ml^{-1} in TA30 was used as matrix. 0-5000 m/z was investigated. Y-axis display signal intensity, and x-axis display mass-over-charge (m/z) value.*
