# Supplementary material for: Advancing lignin analytics via elucidation of linkage progressions in lignin populations
Source: Commun Chem. 2025 Dec 11;9:31. doi: 10.1038/s42004-025-01841-3 (PMC12820315; doi:10.1038/s42004-025-01841-3)
Supplement: Supplementary file 3 — Description of Additional Supplementary Files [file 42004_2025_1841_MOESM3_ESM.docx]

**Description of Additional Supplementary Files:**

**File:** Supplementary Data 1

**Description**: Supplementary Data 1 contains MALDI-TOF files used for LPM creation. Full spectra data files (raw data) and mass lists are provided.

**File:** Supplementary Data 2

**Description**: Supplementary Data 2 contains all NMR files of relevance.
