# Supplementary figures and images for: Advancing lignin analytics via elucidation of linkage progressions in lignin populations
Source: Commun Chem. 2025 Dec 11;9:31. doi: 10.1038/s42004-025-01841-3 (PMC12820315; doi:10.1038/s42004-025-01841-3)

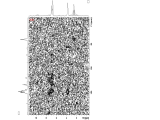

Supplement: Supplementary file 5 — Supplementary Data 2 [file 42004_2025_1841_MOESM5_ESM.zip › HMBC 400 MHz MWL SPRUCE 200 scans/2 (in article)/pdata/1/thumb.png]

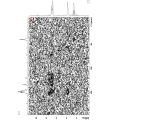

Supplement: Supplementary file 5 — Supplementary Data 2 [file 42004_2025_1841_MOESM5_ESM.zip › HMBC 400 MHz MWL SPRUCE 200 scans/3 (run slightly different)/pdata/1/thumb.png]

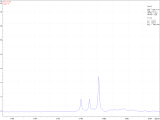

Supplement: Supplementary file 5 — Supplementary Data 2 [file 42004_2025_1841_MOESM5_ESM.zip › HSQC acetylated MWLS 900 MHz 24 scans/1/pdata/1/thumb.png]

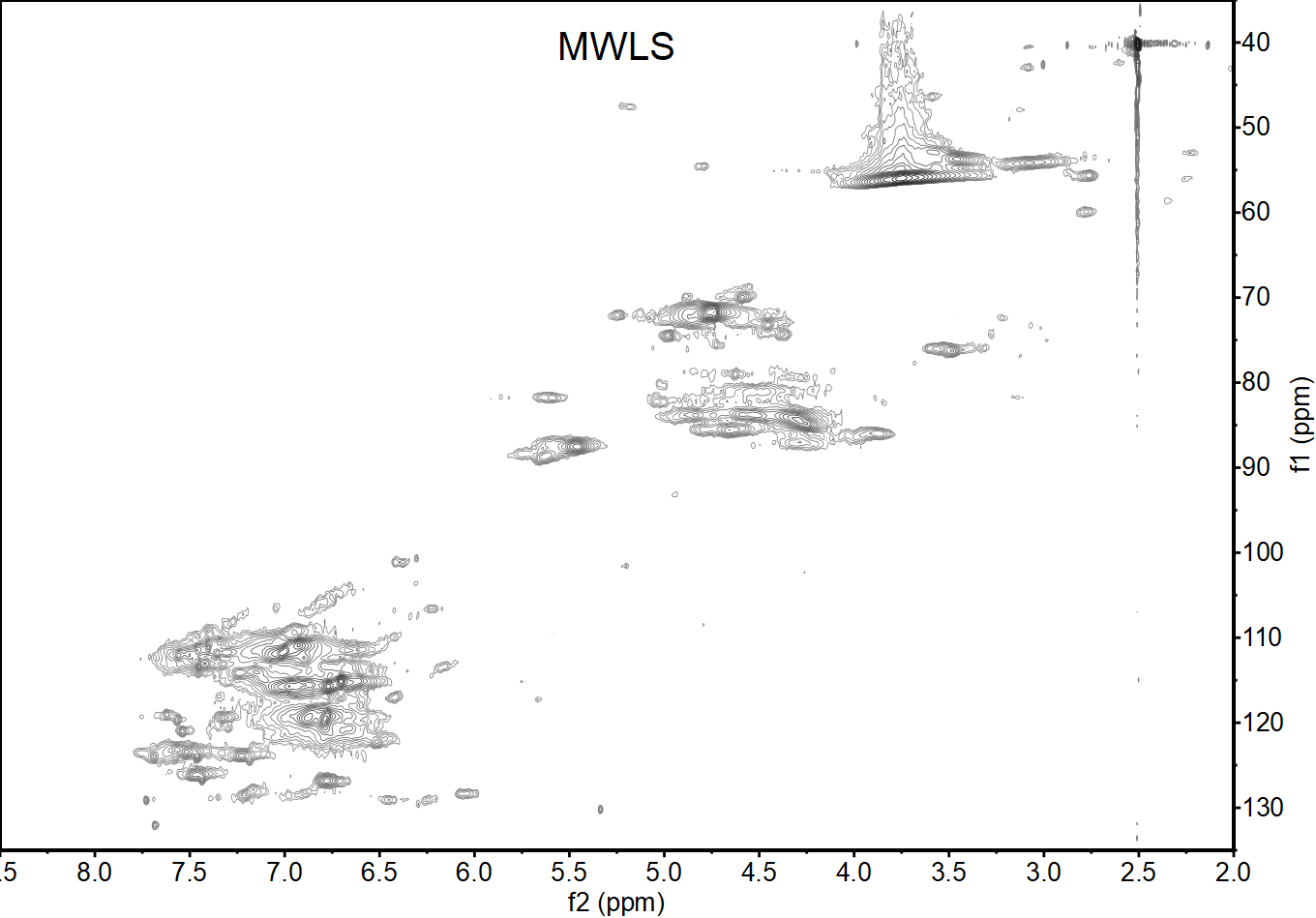

Supplement: Supplementary file 5 — Supplementary Data 2 [file 42004_2025_1841_MOESM5_ESM.zip › HSQC acetylated MWLS 900 MHz 24 scans/2/MWLS.tiff]
